# A Time Series Evaluation of the FAST National Stroke Awareness Campaign in England

**DOI:** 10.1371/journal.pone.0104289

**Published:** 2014-08-13

**Authors:** Darren Flynn, Gary A. Ford, Helen Rodgers, Christopher Price, Nick Steen, Richard G. Thomson

**Affiliations:** 1 Institute of Health and Society, Newcastle University, Newcastle upon Tyne, United Kingdom; 2 Institute for Ageing and Health (Stroke Research Group), Faculty of Medical Sciences, Newcastle University, Newcastle upon Tyne, United Kingdom; 3 Wansbeck General Hospital, Northumbria Healthcare NHS Foundation Trust, Ashington, United Kingdom; CUNY, United States of America

## Abstract

**Objective:**

In February 2009, the Department of Health in England launched the Face, Arm, Speech, and Time (FAST) mass media campaign, to raise public awareness of stroke symptoms and the need for an emergency response. We aimed to evaluate the impact of three consecutive phases of FAST using population-level measures of behaviour in England.

**Methods:**

Interrupted time series (May 2007 to February 2011) assessed the impact of the campaign on: access to a national stroke charity's information resources (Stroke Association [SA]); emergency hospital admissions with a primary diagnosis of stroke (Hospital Episode Statistics for England); and thrombolysis activity from centres in England contributing data to the Safe Implementation of Thrombolysis in Stroke UK database.

**Results:**

Before the campaign, emergency admissions (and patients admitted via accident and emergency [A&E]) and thrombolysis activity was increasing significantly over time, whereas emergency admissions via general practitioners (GPs) were decreasing significantly. SA webpage views, calls to their helpline and information materials dispatched increased significantly after phase one. Website hits/views, and information materials dispatched decreased after phase one; these outcomes increased significantly during phases two and three. After phase one there were significant increases in overall emergency admissions (505, 95% CI = 75 to 935) and patients admitted via A&E (451, 95% CI = 26 to 875). Significantly fewer monthly emergency admissions via GPs were reported after phase three (−19, 95% CI = −29 to −9). Thrombolysis activity per month significantly increased after phases one (3, 95% CI = 1 to 6), and three (3, 95% CI = 1 to 4).

**Conclusions:**

Phase one had a statistically significant impact on information seeking behaviour and emergency admissions, with additional impact that may be attributable to subsequent phases on information seeking behaviour, emergency admissions via GPs, and thrombolysis activity. Future campaigns should be a0ccompanied by evaluation of impact on clinical outcomes such as reduced stroke-related morbidity and mortality.

## Introduction

Stroke is the third leading cause of death worldwide and a major cause of severe adult disability in developed countries [1], [2]. For acute ischaemic stroke, rapid thrombolysis within 4.5 hours can improve the prognosis, with greater benefit from earlier treatment [3], [4]. An expeditious response to stroke symptoms is critical [5]. Many patients present too late to hospital due to a failure, for a variety of reasons, or inability to call emergency services rapidly [5]–[7].

Mass media interventions are promoted as an effective method of improving awareness of health issues and changing behaviour, including encouraging appropriate use of services [8]. A systematic review of 20 evaluations of mass media interventions for a range of conditions reported a positive effect on health services utilisation [8]. Such interventions targeted at stroke have a positive impact on knowledge, including awareness of the need for an emergency response, but with little impact on public behaviour [9].

In February 2009, the Department of Health in England launched the Face, Arm, Speech, and Time (FAST) mass media campaign, to raise public awareness of stroke, specifically its symptoms and the need for an emergency response [10], [11]. An adapted version of the FAST mnemonic (used to screen for the presence of stroke in clinical practice) was used to convey information on three typical stroke symptoms (F = facial weakness, A – arm weakness and S = speech disturbance) and the desired behavioural response (T = time to call emergency services if you recognise any of one of the stroke symptoms). The damage to the brain from stroke was illustrated with an evocative image of a fire rapidly spreading in the head of an older adult depicted. The need for an expeditious response was further emphasised with the aphorism ‘ACT FAST’ along with the key message “The faster you act the more of the person you save”.

Phase one was launched on 9^th^ February 2009 and ran until end of March 2009 with television, press, and radio advertisements. A second phase ran from November 2009 to December 2009; a third phase during February 2010 to March 2010; with a fourth phase during March 2011. Fifth and sixth phases ran from February 2012 to March 2012, and during March 2013. The most recent phase took place during March 2014. Whilst the overall cost of the campaign to date is unclear, and unlikely to be the figure of £105 million in funding over three years quoted in a Wellcome Trust article [12], the costs are not insignificant; the Department of Health stated that the more restricted three month advertising campaign in phase 4 cost £740,000 [13].

Few studies have evaluated the impact of the campaign on behaviour, although face-to-face structured interviews with members of the public in England conducted immediately before and after the first phase identified increases in awareness of stroke signs, the FAST mnemonic, and behavioural intentions to seek emergency medical care for stroke symptoms [7], [14]. A questionnaire survey administered in one mixed urban/rural population in England also reported high-levels of awareness of the campaign (70%) and FAST symptoms (other symptoms, leg weakness and visual loss that were not highlighted in the FAST campaign, were poorly recognised as signs of stroke) [15]. An audit of ambulance trusts in England reportedly attributed an average increase of 50% in absolute numbers of emergency calls categorised as stroke according to paramedics between April to June 2008 (N = 2020) and April to June 2009 (N = 3040) to the campaign [16]. No effect of the campaign on speed of presentation or numbers of patients thrombolysed were reported by single site studies in England [17], [18].

An evaluation (using observational data and modelling) commissioned by the Department of Health attributed a range of impacts on public and professional behaviour to the FAST campaign within the first year: (i) increased numbers of stroke-related emergency calls (55% over the first four months); (ii) increased numbers of patients presenting earlier to hospital (9900) and receiving specialist treatment (2500); (iii) reduced stroke-related morbidity and mortality (640 patients); (iv) gains in quality adjusted life years (2200); and (v) a return on marketing investment of £3.20 for every £1 spent on the campaign [19]. However the time frame and assumptions of the modelling process have not been clearly described.

Attributing these findings to the campaign is difficult since they are cross-sectional surveys [15], clinical outcomes are based on modelling as opposed to objective data [19], or only report changes in summary statistics between pre- and post-intervention periods [14], [16]–[18]. Consequently, they fail to provide an estimate of effect that takes into account the influence of time trend [8]; for example thrombolytic treatment rates in England were already increasing rapidly before the FAST campaign due to service developments [20].

In order to account for the influence of the campaign over and above background time trend, we aimed to evaluate the impact of three consecutive phases of the FAST campaign using objective measures of behaviour at the population level in England with a time series evaluation.

## Methods

### Data sources

An interrupted time series design was used as it is the optimal research design for retrospectively evaluating the impact of the FAST campaign, which also takes into account the influence of background time trend. Three data sources covering the period May 2007 to February 2011 were used to assess the impact of the first, second, and third phases of the FAST campaign: data from the Stroke Association (SA – a registered national charity that provides support and information to stroke survivors and funds research on prevention and treatment of stroke) on resource utilisation [21]; Hospital Episode Statistics (HES) for England (routinely collected information on all patients who receive care from the National Health Service - publicly-funded primary and secondary care services, the majority of which are free to anyone who is legally residing in England) [22]; and data from a monitoring registry set up to audit the safety and efficacy of thrombolysis in the treatment of acute ischaemic stroke in routine settings - the Safe Implementation of Thrombolysis in Stroke UK (SITS-UK) database [23]. The number of SITS-UK hospital sites in England that contributed data to the reported analysis throughout the study period (2007, 2008, 2009, 2010 and up to Feb 2011) was 27.

Data provided by the SA were used to establish the impact of the campaign on information seeking behaviour for stroke: calls to their helpline; visits to their website, including page views; and information materials on stroke dispatched.

HES data for England (finished admission episodes) were used to determine the proportion of overall emergency admissions with a primary diagnosis of stroke admitted via (i) Accident and Emergency (A&E) services – emergency 999 calls, (ii) general practitioner (primary care practitioner) - where members of public contacted a primary care practitioner as the first response to stroke symptoms, rather than initiated an immediate 999 call).; (iii) Bed Bureau, including the Central Bureau (service that collates information on care homes in the UK); (iv) consultant outpatient clinics; and (v) other means, including patients who arrive via the A&E department of another healthcare provider. The following ICD diagnosis codes were aggregated to determine a primary diagnosis of stroke: G45, G46, I61, I63, I64, and I67. Sub-group analyses of emergency admissions via A&E services and emergency admissions via general practitioners were also conducted.

Data from the SITS-UK database [23] from hospitals in England that submitted data to the register throughout the study period (n = 27) were used to assess the impact of the campaign on numbers of patients receiving thrombolytic treatment.

### Statistical analyses

Segmented regression analyses [24] were conducted to establish the following parameters with 95% confidence intervals (CIs): (i) time trends (monthly changes) for data before the campaign (May 2007 to February 2009); (ii) predicted value for outcomes at March 2009 if phase one had not occurred; (iii) any step change in levels for data immediately after phase one (between February 2009 and March 2009); and (iv) magnitude of time trends (monthly changes) for data across March 2009 to October 2009 (corresponding to the period with no campaign activity after phase one), November 2009 to February 2010 (corresponding to the period during the second and third phases), and March 2010 to February 2011 (corresponding to the period of no campaign activity following phase three).

This approach differs to a standard segmented regression analysis where it would be typical to allow for step changes in level for data between October 2009/November 2009 to December 2009, and between January 2010/February 2010 to March 2010. However, in view of the intense compact nature of the initial campaign (and due to the measurement interval of our data we could not model changes during the February 2009 and March 2009) we assumed that the maximum impact on outcomes would occur at the end of March 2009; therefore, it was more appropriate to fit a step change in level only for data between February 2009 and March 2009. The second and third phases occurred over a sufficiently long period to be included as separate segments in our analyses.

The Durbin-Watson statistic (*d*) with lower (*d*l) and upper (*d*u) bounds for critical values at 1% level of significance for regression models with an intercept term [25] was used to establish the presence of autocorrelation of error terms in the regression analyses (i.e. whether consecutive monthly time points were correlated). If *d*>*d*u, or 4-*d*>*d*u then there is no statistical evidence of positive or negative autocorrelation respectively. The inclusion of dummy variables for calendar month into the regression models was undertaken to adjust for seasonality in the datasets. These were conducted as sensitivity analyses, as opposed to main analyses for the following reasons: (i) they constituted 11 of 45 degrees of freedom that may increase the standard errors associated with estimates of effects; and (ii) some variation between months is likely due to FAST activity and thus controlling for calendar months may produce conservative estimates of campaign effects. All analyses were conducted using SPSS version 19.

## Results

**Figure 1:**
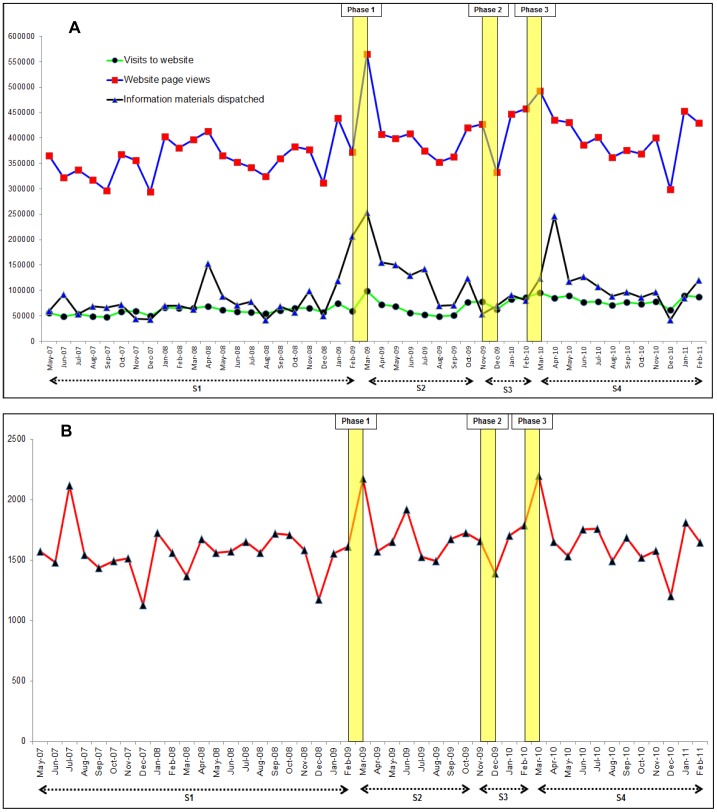
Time series graphs of monthly resource utilisation by the Stroke Association over the study period (May 2007 to February 2011). A: Absolute numbers of website page views, website hits and information materials dispatched by the Stroke Association. B: Absolute numbers of telephone calls received by the Stroke Association helpline. S1: period before the campaign (May 2007 to Feb 2009). S2: period of no campaign activity after phase one of the campaign (Mar 2009 to Oct 2009). S3: period during phases two and three of the campaign (Nov 2009 to Feb 2010). S4: subsequent period with no campaign activity after phase three (Mar 2010 to Feb 2011). Yellow vertical bars represent the time periods for the different phases of campaign activity.

**Figure 2:**
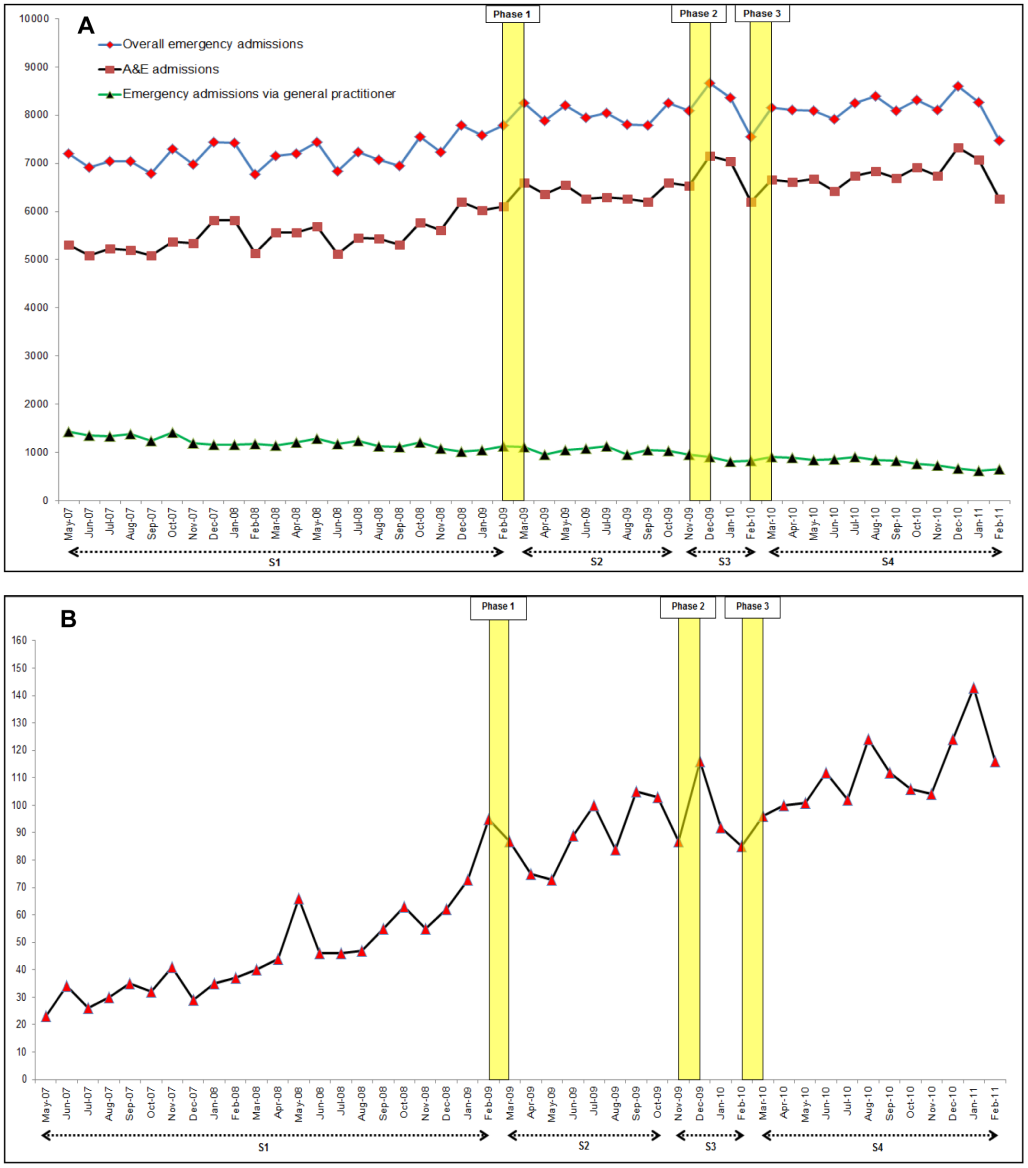
Time series graphs of monthly emergency admissions for stroke and thrombolysis activity over the study period (May 2007 to February 2011). A: Absolute numbers of emergency admissions with a primary diagnosis of stroke in England. B: Absolute numbers of patients receiving thrombolytic treatment in England (centres that submitted data to the SITS register throughout the study period). S1: period before the campaign (May 2007 to Feb 2009). S2: period of no campaign activity after phase one of the campaign (Mar 2009 to Oct 2009). S3: period during phases two and three of the campaign (Nov 2009 to Feb 2010). S4: subsequent period with no campaign activity after phase three (Mar 2010 to Feb 2011). Yellow vertical bars represent the time periods for the different phases of campaign activity.

There was no evidence of autocorrelation in the regression models for the main analyses. The results are presented graphically in figures 1 and 2 and summarised in tables 1 and 2. Regression models adjusted for seasonality are shown in Tables S1 and S2.

**Table 1:** Interval estimates of segmented regression coefficients for changes in data for different time periods.

	S1: before the campaign (May 07 to Feb 09)			Predicted mean at March 2009			Change in level immediately after phase one (Feb 09 to Mar 09)			S2: period of no campaign activity after phase one (Mar 09 to Oct 09)			S3: period during phases two and three of the campaign (Nov 09 to Feb 10)			S4: period with no campaign activity after phase three (Mar 10 to Feb 11)		
Measure	Trend	95% CI		Mean	95% CI		Mean	95% CI		Trend	95% CI		Trend	95% CI		Trend	95% CI	
SA: website hits	613	−22	1248	66387	58051	74722	10030	−4341	24401	−2550	−5014	−85	7553	3729	11378	−1116	−2518	287
SA: webpage views	2043	−972	5058	381589	341991	421188	82114	13845	150384	−13875	−25582	−2167	20946	2779	39113	−6357	−13020	305
SA: information materials	2372	−170	4913	106099	72717	139480	101081	43530	158632	−20970	−30840	−11101	22362	7048	37677	−5433	−11050	183
SA: calls to helpline	−2	−16	12	1538	1356	1719	339	26	652	−48	−101	6	72	−11	155	−27	−57	4
HES: overall admissions	26	7	45	7510	7260	7759	505	75	935	13	−60	87	4	−111	118	1	−41	43
HES: A&E admissions	35	16	54	5916	5669	6162	451	26	875	23	−49	96	27	−86	140	18	−23	60
HES: admissions via GP	−14	−19	−10	1046	986	1106	35	−68	138	−13	−31	4	−21	−49	6	−19	−29	−9
SITS England: Thrombolysis activity	2	2	3	73	65	82	5	−10	19	3	1	6	−2	−6	2	3	1	4

Figures are absolute numbers.

Predicted mean at March 2009 are predicted values for data if phase 1 had not occurred.

Change in level - step change in levels for data immediately after phase one (between February 2009 and March 2009).

Trends refer to monthly changes in data.

SA: Stroke Association; HES (Hospital Episode Statistics); A&E (accident and emergency); SITS (Safe Implementation of Thrombolysis in Stroke).

**Table 2:** Summary of statistically significant (p<0.5) changes in data for different time periods.

Data Source/Measure	S1: Trend before the campaign (May 07 to Feb 09)	Predicted mean at March 2009	Change in level immediately after phase one (Feb 09 to Mar 09)	S2: Trend for period of no campaign activity after phase one (Mar 09 to Oct 09)	S3: Trend for period during phases two and three of the campaign (Nov 09 to Feb 10)	S4: Trend for period with no campaign activity after phase three (Mar 10 to Feb 11)
SA: Website hits	NS increase	66387	NS increase	Significant decline	Significant increase	NS decline
SA: Website page views	NS increase	381589	Significant increase	Significant decline	Significant increase	NS decline
SA: Information materials	NS increase	106099	Significant increase	Significant decline	Significant increase	NS decline
SA: Calls to helpline	NS decline	1538	Significant increase	NS decline	NS increase	NS decline
HES: Overall emergency admissions	Significant increase	7510	Significant increase	NS increase	NS increase	NS increase
HES: A&E admissions	Significant increase	5916	Significant increase	NS increase	NS increase	NS increase
HES: Emergency admission via GP	Significant decline	1046	NS increase	NS decline	NS decline	Significant decline
SITS England: Thrombolysis activity	Significant increase	54	NS increase	Significant increase	NS decline	Significant increase

Figures for predicted mean at March 2009 are absolute numbers – predicted values if phase 1 had not occurred.

Change in level - step change in levels for data immediately after phase one (between February 2009 and March 2009).

Trends refer to monthly changes in data.

SA: Stroke Association; HES (Hospital Episode Statistics); A&E (accident and emergency); SITS (Safe Implementation of Thrombolysis in Stroke).

NS = non-significant at p<0.05.

### The Stroke Association (SA)

Figure 1 (panels A and B) shows data on resource utilisation for the SA: website hits (N = 3104722), website page views (N = 17668701), calls to their helpline (N = 74428) and information materials dispatched (N = 4463983). There were no statistically significant changes in trends for these outcomes before the campaign.

With the exception of website visits, significant changes in level between February 2009 and March 2009 were identified for webpage views (82144, 95% CI = 13845 to 150384), information materials dispatched (101081, 95% CI = 43530 to 158632), and calls to the SA helpline (339, 95% CI = 26 to 652). Significant monthly decreases in website visits (−2550, 95% CI = −5014 to −85), webpage views (−13875, 95% CI = −25582 to −2167), and information materials dispatched (−20970, 95% CI = −30840 to −11101) occurred during the period of no campaign activity following phase one. These outcomes increased significantly during the second and third phases of the campaign. Changes in numbers of calls to the SA helpline across subsequent phases of the campaign were not statistically significant.

### HES for England

Figure 2 (panel A) shows HES data (N = 353305) for overall emergency admissions for stroke, and for emergency admissions via A&E (n = 280338) and general practitioners (n = 48019). Statistically significant increases over 22 months before the campaign were identified for overall emergency admissions (26 patients per month; 95% CI = 7 to 45) and A&E admissions (35 patients per month, 95% CI = 16 to 54), with significant increases in levels between February 2009 and March 2009 (FAST Phase one) for both overall (505 patients, 95% CI = 75 to 935) and A&E admissions (451 patients, 95% CI = 26 to 875). Subsequent increases for these outcomes immediately after the first phase, during the second and third phases, and after the third phase were not significant.

Emergency admissions via general practitioners were declining during the period before the campaign (−14 patients per month; 95% CI = −19 to −10). Changes for this mode of emergency admission between February 2009 and March 2009, after the first phase, and during the second and third phases were not significant. However, a significant decrease was identified during the period of no campaign activity after phase three (−19 patients per month, 95% CI = −29 to −9).

### SITS-England

Figure 2 (panel B) shows the number of patients in England receiving thrombolysis from 27 participating hospitals in England that submitted data to the SITS-UK database throughout the observation period (N = 3450). There was a significant monthly increase in thrombolysis activity before the campaign (2 patients per month; 95% CI = 2 to 3), with a non-significant increase in level between February 2009 and March 2009. During the period of no campaign activity after phase one, there was a significant increase in thrombolysis activity (3 patients per month, 95% CI = 1 to 6), followed by a monthly non-significant decline during the second and third phases. A significant increase in thrombolysis was also identified during the period of no campaign activity after phase three (3 patients per month, 95% CI = 1 to 4).

### Results adjusted for seasonality

Adjusting for variation between calendar months in regression models (Tables S1 and S2) resulted in the change in levels between February 2009 and March 2009 for information materials dispatched by the SA and calls to their helpline no longer being statistically significant. There were no longer significant changes in trends for webpage views and information materials dispatched by the SA after phase one and during the second and third phases. Decreased hits on the SA website were also no longer significant after phase one.

Adjusted regression models did not alter the pattern of statistically significant results for emergency admissions or thrombolysis activity; although the magnitudes of effects were decreased (trends before the campaign for emergency admissions) or increased (emergency admissions and webpage views between February 2009 and March 2009, and thrombolysis activity after the first phase).

## Discussion

Statistically significant time trends (monthly increases) before the campaign were reported for emergency hospital admissions for stroke (overall and A&E) and thrombolysis activity from hospitals in England that submitted data to the SITS-UK database, but not for access to information about stroke from the SA. There was also a significant monthly decline before the campaign in numbers of emergency hospital admissions for stroke via general practitioners (GPs) - where the public contacted a primary care practitioner as the first response to stroke symptoms, rather than initiated an immediate 999 call. These underlying trends may in part be explained by (i) the publication of the National Stroke Strategy in December 2007 [10], with subsequent national media attention; (ii) discussions and preparation pertaining to implementation of the National Stroke Strategy within stroke services at the regional level across England; (iii) additional factors influencing the screening of patients for stroke by paramedics (ambulance service national Care Quality Commission audit, and the proposal for emergency response times for suspected stroke occurring less than three hours prior to a 999 call to be changed from category B [serious, but not immediately life-threatening – response time 20 minutes] to a category A [life threatening – response time 8 minutes]) connected with the National Stroke Strategy [26], [27]); and (iv) service development with regards to thrombolytic treatment [20].

We found evidence of increased public access to stroke-related information (website hits, webpage views, information materials dispatched by the SA, and calls to their helpline), increased overall/A&E emergency admissions, decreased emergency admissions via GPs, and increased thrombolysis activity that were attributable to the FAST campaign over and above underlying trends. Although adjusted analyses indicated that the SA datasets may have been influenced by seasonality, emergency hospital admissions for stroke (overall and via A&E) increased significantly immediately after the initial phase, whereas emergency hospital admissions via GPs and thrombolysis activity showed statistically significant decreases and increases respectively after the third phase of the campaign. In contrast to a retrospective audit of stroke patients presenting to a single hospital in England [17], we found a statistically significant increase in thrombolysis activity.

Our findings provide evidence of an enduring impact of the campaign in England on public behaviour (i.e. increased awareness of stroke and more people arriving at secondary care, due to fewer people contacting GPs as the first response to stroke symptoms) and greater numbers of appropriate patients with stroke symptoms arriving at specialist centres within the stroke onset time to treatment window for evidence-based treatments such as thrombolysis.

In contrast to our findings, a time series evaluation of a similar FAST campaign rolled out in Ireland during 2010 was not shown to have a sustained impact on emergency department attendance [28]. Mass media campaigns targeting stroke in the Czech Republic [29] and the USA [30] have also met with limited success.

The reasons for the differences between our findings, and evaluations of other campaigns are complex and multi-factorial, and as previously noted their dual nature (public and professional) makes it difficult to elucidate active components that might explain any reported impact [9]. It has been posited that effectiveness of mass media campaigns targeting stroke may be enhanced by spend on media, media mix, and key messages [14]. Message content (e.g. possibility of acute treatments such as thrombolysis [31]) and the type of language (used by patients/bystanders versus professional) used to describe common stroke symptoms [31] may also impact on effectiveness. Furthermore, failure to present details of theoretically grounded development and piloting of the interventions [9] further compounds identification of the optimal active ingredients of effective campaigns.

The message content of FAST in England did not focus on overcoming barriers to performing the desired response (call an ambulance) or adequately address response efficacy (i.e., beliefs associated with executing the desired response by explicitly referring to the availability of an effective emergency treatment such as thrombolysis). Additional impact may be observed with the addition of these components to the message content.

The measures of emergency admissions and thrombolysis activity directly relate to impact on patients with incident stroke and subsequent outcomes, whereas measures of information seeking (access to information resources from the SA) is more indicative of behaviour of people who wish to know if they (or their services) should have done something different when they (or others) experienced a stroke.

These findings are important, but should also be interpreted with some caution. Time series designs increase the confidence that estimates of effect can be attributed to the intervention [32]. Nevertheless, there is always a chance that patterns in these observational data were due to unobserved confounding variables; not all sites in England enter data onto the SITS database and it is possible that thrombolysis activity at these sites had different trends. The number of hospital sites in England that contributed data to the SITS-UK database during 2007, 2008, 2009, 2010 and 2011 were 50, 84, 112, 124 and 89 respectively. However, analyses based on 27 centres contributing thrombolysis activity data throughout the observation period enable a more accurate estimate of an effect of the campaign on public behaviour (changes in thrombolysis activity in other centres might have been due to internal hospital factors). Furthermore, in line with national guidelines [27] the proposed change in emergency 999 response times for suspected stroke was implemented in March/April 2009 (stroke occurring less than three hours prior to a 999 call was changed to a category A (life threatening – response time 8 minutes), which may have led to increased screening of patients for stroke by 999 dispatchers and paramedics, which resulted in more patients reaching hospital within the timeframe for thrombolysis.

Overall, our analyses show a statistically significant impact of phase one of the FAST campaign over and above underlying time trends on information seeking behaviour on stroke (via the SA data) and emergency admissions for stroke (overall and cases admitted via A&E). Our analyses also provide evidence that subsequent phases supported the maintenance and augmented the impact of the initial phase on information seeking behaviour, emergency hospital admissions via GPs and thrombolysis activity. The statistically significant impact on the latter two outcomes after the third phase is likely due to the close proximity of the second and third phases, in effect a ‘double-dose’ of the intervention.

Nevertheless, our findings for magnitudes of the direct effects for phase one and subsequent phases in terms of their clinical significance are at best modest, indicating that the clinical significance and cost-effectiveness of the campaign was substantially less than that modelled by a previous evaluation [19].

Analyses exploring the impact of time from onset of stroke symptoms to time to arrival at hospital would be important (as earlier treatment with thrombolysis increases likelihood of a good outcome), but data on onset time of the stroke are not available in any routine system. Similarly, data reporting on changes in time from arrival to hospital to time of receipt of thrombolysis (door to needle times) are also not routinely available; however door to needle time is not relevant to the aim of the campaign, which targeted emergency response to symptoms of stroke by members of the public.

The underlying assumption is that if more people with stroke symptoms arrive rapidly at secondary care that outcomes will improve through the increased use of evidence-based treatment such as thrombolysis (as identified by our analysis of emergency admissions and thrombolysis activity, although as stated above not all sites in England enter data onto the SITS database). Using the figures for the statistically significant step change increase (FAST Phase one) between February 2009 and March 2009 for overall emergency stroke admissions in England (505 patients, 95% CI = 75 to 935) as an exemplar allows a very conservative estimate (that takes no account of patients arriving at hospital more rapidly and being treated earlier within the 4.5 hour time window for thrombolysis and the resultant increased likelihood of benefit from treatment) of clinical impact on the campaign. Assuming 85% of these cases will be acute ischaemic stroke (505×0.85 = 429 patients), and further assuming a thrombolysis rate of 12% [33] (429×0.12) then 51 additional patients may have received thrombolysis. The mean number needed to treat for a good outcome (independence) is 8 [3], which equates to an additional (51/8) 6 patients in England who may have had good outcomes from treatment. Applying the confidence interval ranges for the step change between February 2009 and March 2009 results in a lower and upper conservative estimate of 1 and 12 additional patients who may have received a good outcome from treatment with thrombolysis.

The conclusions are strengthened by the inclusion of three independent data sources. Segmented regression analyses enabled us to estimate the effect of FAST on multiple objective measures of behaviour, which further highlights the importance of robust evaluation of mass media interventions [8], [9]. Reliance on findings from evaluative studies reporting on awareness and behavioural intentions, small single site studies, and modelling techniques can lead to false confidence in the effectiveness of this type of complex intervention on health outcome benefits and cost-effectiveness. The findings from our study emphasise the importance of robust evaluation and this should extend to all mass media campaigns that represent significant investment of resources.

The need for continuous advertising to sustain public stroke awareness has been highlighted previously [34], [35]. Additional evaluation studies of subsequent phases of the FAST campaign (phase four during March 2011, with fifth, sixth and seventh phases rolled out during February and March 2012, March 2013 and March 2014 respectively) would be helpful to establish if subsequent phases yielded any additional statistically significant impact or sustain the impact of previous phases on public behaviour.

Theory-based interventions targeting the entire range of stroke symptoms, barriers to calling an ambulance, and specific information on the availability of effective secondary care treatments such as thrombolysis, may be warranted to further augment the impact of the campaign message on clinical outcomes [36].

In order to elucidate causal mechanisms that sustain the impact of mass media interventions on public behaviour, designers, policy makers, clinicians and researchers in the field of stroke (and other disciplines where the design of mass media interventions are planned) should utilise appropriate theory and adhere to a structured development process, and undertake robust evaluation before, during and after the roll-out phases using objective measures of behaviour at the population level.

## Conclusions

We used the optimal research design to retrospectively evaluate the impact of the FAST campaign, which enables analysis of impact over and above any underlying time trend (interrupted time series evaluation of appropriately available behavioural data at the population level in England). There was an initial statistically significant impact of phase one on information seeking behaviour of the public about stroke and emergency admissions for incident stroke, with additional statistically significant impact on information seeking behaviour, emergency hospital admissions (reduction in inappropriate care seeking behaviour of the public, i.e. contacting a primary care practitioner as the first response to stroke symptoms), and thrombolysis activity (arguably the most important measure of impact directly related to clinical outcomes). The FAST campaign has been promoted internationally as a great success [19], [37]; although our analysis suggests more modest effects in terms of its clinical impact. Consequently, the clinical impact and cost-effectiveness of the FAST campaign in England remains unclear and future campaigns should be accompanied by formal evaluation of impact on clinical outcomes.

## Supporting Information

Table S1 **Interval estimates of segmented regression coefficients for changes in data for different time periods (adjusted for seasonality).** Figures are absolute numbers. Predicted mean at March 2009 are predicted values for data if phase 1 had not occurred. Change in level - step change in levels for data immediately after phase one (between February 2009 and March 2009). Trends refer to monthly changes in data. SA: Stroke Association; HES (Hospital Episode Statistics); A&E (accident and emergency); SITS (Safe Implementation of Thrombolysis in Stroke).
(DOCX)

Table S2 **Summary of statistically significant (p<0.5) changes in data for different time periods (adjusted for seasonality).** Figures for predicted mean at March 2009 are absolute numbers – predicted values if phase 1 had not occurred. Change in level - step change in levels for data immediately after phase one (between February 2009 and March 2009). Trends refer to monthly changes in data. SA: Stroke Association; HES (Hospital Episode Statistics); A&E (accident and emergency); SITS (Safe Implementation of Thrombolysis in Stroke). NS = non-significant at p<0.05.
(DOCX)
