# Supplementary material for: A Time Series Evaluation of the FAST National Stroke Awareness Campaign in England
Source: PLoS One. 2014 Aug 13;9(8):e104289. doi: 10.1371/journal.pone.0104289 (PMC4131890; doi:10.1371/journal.pone.0104289)
Supplement: Table S1 — Interval estimates of segmented regression coefficients for changes in data for different time periods (adjusted for seasonality). Figures are absolute numbers. Predicted mean at March 2009 are predicted values for data if phase 1 had not occurred. Change in level - step change in levels for data immediately after phase one (between February 2009 and March 2009). Trends refer to monthly changes in data. SA: Stroke Association; HES (Hospital Episode Statistics); A&E (accident and emergency); SITS (Safe Implementation of Thrombolysis in Stroke). (DOCX) [file pone.0104289.s001.docx]

Table S1. Interval estimates of segmented regression coefficients for changes in data for different time periods (adjusted for seasonality)

|  | **S1: before the campaign** (May 07 to Feb 09) | | | **Predicted mean at March 2009** | | | **Change in level immediately after phase one** (Feb 09 to Mar 09) | | | **S2: period of no campaign activity after phase one** (Mar 09 to Oct 09) | | | **S3: period during phases two and three of the campaign** (Nov 09 to Feb 10) | | | **S4: period with no campaign activity after phase three** (Mar 10 to Feb 11) | | |
| --- | --- | --- | --- | --- | --- | --- | --- | --- | --- | --- | --- | --- | --- | --- | --- | --- | --- | --- |
| **Measure** | **Trend** | **95% CI** | | **Mean** | **95% CI** | | **Mean** | **95% CI** | | **Trend** | **95% CI** | | **Trend** | **95% CI** | | **Trend** | **95% CI** | |
| SA: website visits | 441 | -10 | 893 | 69013 | 58971 | 79056 | 5810 | -6168 | 17788 | -1025 | -3139 | -1089 | 5152 | 2012 | 8293 | -607 | -1680 | 467 |
| SA: webpage views | 1384 | -303 | 3071 | 394781 | 357284 | 432278 | 51934 | 7209 | 96658 | -4560 | -12454 | -3334 | 6539 | -5187 | 18266 | -3180 | -7188 | 827 |
| SA: information materials | 2463 | -40 | 4966 | 176538 | 120900 | 232175 | 57792 | -8570 | 124154 | -11326 | -23039 | 387 | 8618 | -8783 | 26018 | -1841 | -7787 | 4105 |
| SA: calls to helpline | -1 | -13 | 11 | 1507 | 1235 | 1780 | 223 | -102 | 547 | -24 | -82 | 33 | 40 | -46 | 125 | -18 | -47 | 11 |
| HES: overall admissions | 24 | 10 | 38 | 7397 | 7083 | 7712 | 597 | 222 | 973 | -5 | -72 | 61 | 31 | -67 | 130 | -6 | -40 | 27 |
| HES: A&E admissions | 30 | 18 | 41 | 5809 | 5553 | 6065 | 600 | 295 | 906 | 5 | -49 | 59 | 47 | -33 | 128 | 10 | -18 | 37 |
| HES: admissions: GP | -12 | -15 | -8 | 1058 | 975 | 1142 | -32 | -131 | 67 | -7 | -25 | 10 | -26 | -52 | 1 | -16 | -25 | -7 |
| SITS England: Thrombolysis activity | 2 | 2 | 3 | 73 | 56 | 90 | 1 | -20 | 21 | 4 | 1 | 8 | -3 | -8 | 2 | 3 | 1 | 5 |

Figures are absolute numbers

Predicted mean at March 2009 are predicted values for data if phase 1 had not occurred

Change in level - step change in levels for data immediately after phase one (between February 2009 and March 2009)

Trends refer to monthly changes in data

SA: Stroke Association; HES (Hospital Episode Statistics); A&E (accident and emergency); SITS (Safe Implementation of Thrombolysis in Stroke)
