# Supplementary material for: A Time Series Evaluation of the FAST National Stroke Awareness Campaign in England
Source: PLoS One. 2014 Aug 13;9(8):e104289. doi: 10.1371/journal.pone.0104289 (PMC4131890; doi:10.1371/journal.pone.0104289)
Supplement: Table S2 — Summary of statistically significant (p<0.5) changes in data for different time periods (adjusted for seasonality). Figures for predicted mean at March 2009 are absolute numbers – predicted values if phase 1 had not occurred. Change in level - step change in levels for data immediately after phase one (between February 2009 and March 2009). Trends refer to monthly changes in data. SA: Stroke Association; HES (Hospital Episode Statistics); A&E (accident and emergency); SITS (Safe Implementation of Thrombolysis in Stroke). NS = non-significant at p<0.05. (DOCX) [file pone.0104289.s002.docx]

Table S2. Summary of statistically significant (p<0.5) changes in data for different time periods (adjusted for seasonality)

| **Data Source / Measure** | **S1: Trend before the campaign** (May 07 to Feb 09) | **Predicted mean at March 2009** | **Change in level immediately after phase one** (Feb 09 to Mar 09) | **S2: Trend for period of no campaign activity after phase one** (Mar 09 to Oct 09) | **S3: Trend for period during phases two and three of the campaign** (Nov 09 to Feb 10) | **S4: Trend for period with no campaign activity after phase three** (Mar 10 to Feb 11) |
| --- | --- | --- | --- | --- | --- | --- |
| SA: Website hits | NS increase | 69013 | NS increase | NS decline | Significant increase | NS decline |
| SA: Website page views | NS increase | 394781 | Significant increase | NS decline | NS increase | NS decline |
| SA: Information materials | NS increase | 176538 | NS increase | NS decline | NS increase | NS decline |
| SA: Calls to helpline | NS decline | 1507 | NS increase | NS decline | NS increase | NS decline |
| HES: Overall emergency admissions | Significant increase | 7397 | Significant increase | NS decline | NS increase | NS decline |
| HES: A&E admissions | Significant increase | 5809 | Significant increase | NS increase | NS increase | NS increase |
| HES: Emergency admissions: GP | Significant decline | 1058 | NS decline | NS decline | NS decline | Significant decline |
| SITS England: Thrombolysis activity | Significant increase | 57 | NS increase | Significant increase | NS decline | Significant increase |

Figures for predicted mean at March 2009 are absolute numbers – predicted values if phase 1 had not occurred

Change in level - step change in levels for data immediately after phase one (between February 2009 and March 2009)

Trends refer to monthly changes in data

SA: Stroke Association; HES (Hospital Episode Statistics); A&E (accident and emergency); SITS (Safe Implementation of Thrombolysis in Stroke)

NS = non-significant at p < 0.05
